# Improving Air Quality by Nitric Oxide Consumption of Climate-Resilient Trees Suitable for Urban Greening

**DOI:** 10.3389/fpls.2020.549913

**Published:** 2020-09-29

**Authors:** Jiangli Zhang, Andrea Ghirardo, Antonella Gori, Andreas Albert, Franz Buegger, Rocco Pace, Elisabeth Georgii, Rüdiger Grote, Jörg-Peter Schnitzler, Jörg Durner, Christian Lindermayr

**Affiliations:** ^1^ Institute of Biochemical Plant Pathology, Helmholtz Zentrum München, Neuherberg/Munich, Germany; ^2^ College of Life Sciences, Henan Normal University, Xinxiang, China; ^3^ Research Unit Environmental Simulation, Institute of Biochemical Plant Pathology, Helmholtz Zentrum München, Neuherberg/Munich, Germany; ^4^ Department of Agriculture, Food, Environment, and Forestry (DAGRI), University of Florence, Florence, Italy; ^5^ Department of Biology, Agriculture and Food Sciences, Institute for Sustainable Plant Protection, The National Research Council of Italy (CNR), Florence, Italy; ^6^ Institute of Meteorology and Climate Research — Institute of Atmospheric Environmental Research, Karlsruhe Institute of Technology, Garmisch-Partenkirchen, Germany; ^7^ Institute of Research on Terrestrial Ecosystems (IRET), National Research Council (CNR), Porano, Italy; ^8^ Chair of Biochemical Plant Pathology, Technische Universität München, Freising, Germany

**Keywords:** nitric oxide, nitrogen dioxide, ^15^N, phytoglobin, air pollution mitigation, urban trees

## Abstract

Nitrogen oxides (NO_x_), mainly a mixture of nitric oxide (NO) and nitrogen dioxide (NO_2_), are formed by the reaction of nitrogen and oxygen compounds in the air as a result of combustion processes and traffic. Both deposit into leaves via stomata, which on the one hand benefits air quality and on the other hand provides an additional source of nitrogen for plants. In this study, we first determined the NO and NO_2_ specific deposition velocities based on projected leaf area (s*V*_d_) using a branch enclosure system. We studied four tree species that are regarded as suitable to be planted under predicted future urban climate conditions: *Carpinus betulus*, *Fraxinus ornus*, *Fraxinus pennsylvanica* and *Ostrya carpinifolia*. The NO and NO_2_ *sV_d_* were found similar in all tree species. Second, in order to confirm NO metabolization, we fumigated plants with ^15^NO and quantified the incorporation of ^15^N in leaf materials of these trees and four additional urban tree species (*Celtis australis*, *Alnus spaethii*, *Alnus glutinosa*, and *Tilia henryana*) under controlled environmental conditions. Based on these ^15^N-labeling experiments, *A. glutinosa* showed the most effective incorporation of ^15^NO. Third, we tried to elucidate the mechanism of metabolization. Therefore, we generated transgenic poplars overexpressing *Arabidopsis thaliana* phytoglobin 1 or 2. Phytoglobins are known to metabolize NO to nitrate in the presence of oxygen. The ^15^N uptake in phytoglobin-overexpressing poplars was significantly increased compared to wild-type trees, demonstrating that the NO uptake is enzymatically controlled besides stomatal dependence. In order to upscale the results and to investigate if a trade-off exists between air pollution removal and survival probability under future climate conditions, we have additionally carried out a modeling exercise of NO and NO_2_ deposition for the area of central Berlin. If the actually dominant deciduous tree species (*Acer platanoides*, *Tilia cordata*, *Fagus sylvatica*, *Quercus robur*) would be replaced by the species suggested for future conditions, the total annual NO and NO_2_ deposition in the modeled urban area would hardly change, indicating that the service of air pollution removal would not be degraded. These results may help selecting urban tree species in future greening programs.

## Introduction 

Urban air is posing a risk to health in most parts of the world, with emissions from industrial processes, residential heating, and heavy traffic based on fossil fuels being the principal causes. This results in high levels of particles, nitrogen oxides (NO_x_), and other dangerous compounds.

Particularly NO_x_, which is formed by the reaction of nitrogen and oxygen compounds as a result of combustion processes, is a pollutant of great concern since it is directly related to cardiovascular diseases and respiratory malfunctions (Mannucci et al., 2015) as well as being a precursor for ozone formation (Sillman, 1999). Additionally, they have also been found to increase the allergenicity of pollen (Zhao et al., 2016). In areas with heavy vehicle traffic, such as in large cities and conurbations, the amount of NO_x_ emitted as an air pollutant into the lower troposphere is significant, and the resulting concentrations often exceed national regulation. For instance, in 2017, around 10% of all the air quality monitoring stations in Europe recorded average annual concentrations above the annual limit value of 40 μg m^-3^ (EEA, 2019). Plants can play an important role in mitigating the NO_x_ related damages on health and environment because their large surface represents efficient “sinks” for air pollutants (Hill, 1971). In cities, the air phytoremediation abilities combined with other ecosystem services of trees (e.g., mitigate air temperature extremes) give urban greening the potential to improve human health while mitigating the effects of climate change (Salmond et al., 2016; Kabisch and van den Bosch, 2017). Plants remove gaseous air pollutants such as NO_x_ and ozone mainly by uptake through the stomata of leaves, although some gaseous compounds may also be deposited on the plant surface (Elkiey et al., 1982; Jud et al., 2016). The ability to absorb NO_2_ has been reported for a variety of plant species, including many tree species, such as loblolly pine (*Pinus taeda*), white oak (*Quercus alba*), silver birch (*Betula pendula*), European beech (*Fagus sylvatica*), pedunculate oak (*Quercus robur*), holm oak (*Quercus ilex*), California oak (*Quercus agrifolia*), Scots pine (*Pinus sylvestris*), and Norway spruce (*Picea abies*) (Rogers et al., 1979; Geßler et al., 2002; Eller and Sparks, 2006; Chaparro-Suarez et al., 2011; Breuninger et al., 2013; Delaria et al., 2018). NO_2_ deposition is influenced by stomata aperture, nitrogen status, leaf development and -age, photosynthetic rate, and the position of leaves within the plant canopy (Morikawa et al., 1998; Sparks et al., 2001; Takahashi et al., 2005; Hu and Sun, 2010). Thus, a clear difference between tree species and dependence on vitality can be expected. In contrast to NO_2_ deposition, studies on NO uptake by plants are scarce in the literature. Nevertheless, measurements of atmospheric NO levels in the presence of horticultural crops, including lettuce, strawberry, apple, and banana, showed a significant decrease in atmospheric NO concentrations, indicating the ability of these plants to absorb NO (Hanson and Lindberg, 1991; Soegiarto et al., 2003).

If NO_x_ is taken up through the stomata, it needs to be further processed or deposited into the plant structure. In fact, evidence exists that various enzymes have the ability to metabolize NO_x_. For example, phytoglobins (PGBs) are proteins regarded as important for the nitrogen metabolisms and are ubiquitously distributed in plants (Becana et al., 2020). These proteins play a major role in regulating many biological processes, such as normal growth and development, hypoxic stress, symbiotic nodulation and nitrogen fixation, and are activated in response to low mineral nutrient status and abiotic stress (Hebelstrup et al., 2006; Mira et al., 2016; Mira et al., 2017; Shankar et al., 2018; Becana et al., 2020; Berger et al., 2020). Particularly, PGBs can oxidize NO to nitrate during hypoxic stress, which is called the PGB/NO cycle (Igamberdiev and Hill, 2004; Igamberdiev et al., 2006; Becana et al., 2020). In previous publications on *Arabidopsis* and barley, we reported on the ability of PGBs (Kuruthukulangarakoola et al., 2017; Zhang et al., 2019) to fix atmospheric NO and incorporate N into the nitrogen metabolism of the plants. Atmospheric nitrogen supply has been formerly regarded as gaseous nitrogen fixation or ammonia uptake only in connection with microbial or fungal associations (Granhall and Lindberg, 1980; Papen et al., 2002). This new NO fixation process seems to be a new pathway in this cycle, which can potentially play an important role within the whole nitrogen cycle, which is essential for building up proteins, nucleic acids, chlorophyll and many other organic compounds. Although the ability for NO_x_ uptake may be ubiquitous in plants, the actual uptake capacity of different species is likely to vary (Takahashi et al., 2005).

In the near future, tree species composition in urban areas is likely to change towards climate-change resilient species, which can cope with increases in intensity, frequency, and severity of abiotic stresses (Burley et al., 2019). In particular, drought and heat resistance are primary selection criteria for urban greening programs (e.g., Roloff et al., 2009). Therefore, stress-tolerant species such as *C. betulus*, *F. ornus*, *F. pennsylvanica*, *O. carpinifolia*, *C. australis*, *Alnus x spaethii*, *A. glutinosa*, and *T. henryana* are currently being proposed (e.g., Böll, 2017; Dickhaut and Eschenbach, 2019). However, it is known that different tree species have different pollution removal capacities that are related to various leaf traits that influence deposition velocity and to their stomatal behavior in response to drought (Grote et al., 2016). The ability to process NO_x_ may thus be a further trait that influences the uptake of gaseous nitrogen compounds. Particularly trees that are considered suitable under future environmental conditions and which, therefore, might have a reduced stomatal conductance adapted to high temperatures and low water supply, could be assumed to have less NO_x_ removal capacity. Therefore, tree species that are selected to withstand increasing heat and drought stress in urban areas need to be checked for their ability to provide the same degree of ecosystem services, i.e. air pollution removal.

To provide a quantitative estimate of pollution removal of current and potential future tree species, we determined the deposition rates of NO and NO_2_ in tree species that are regarded as suitable candidates for urban trees under future climatic conditions in Central Europe (Böll, 2017; Böll, 2018a; Böll, 2018b). Moreover, we used gas exchange measurements and followed the capacity of NO uptake and metabolization in eight different tree species fumigated with ^15^NO under controlled environmental conditions. Additionally, we demonstrated that the NO uptake could be enhanced in trees by introducing the *Arabidopsis* phytoglobin 1 and 2 (*AtPGB1, AtPBG2*) genes into poplars. Finally, using the newly determined deposition rates, we compared the potential NO_x_ removal for a scenario that assumes a high abundance of tree species proposed for adaptation to climate change conditions with the removal capacities of the current urban tree distribution. This should indicate potential changes in NOx removal due to the selection of these species in future urban planning under “real world conditions”. For this exercise, we use state-of-the-art calculation processes, parameters from literature for the current tree species, and the boundary conditions for a Metropolitan area, Central Berlin.

## Materials and Methods 

### Plants Material

All plant species with altered PGB expression used in this study are listed in **Table 1**. *Arabidopsis thaliana* (Columbia-0) with overexpressing class 1 PGB (*AtPgb1+*) or class 2 *PGB* (*AtPgb2+*), as well as plants with reduced (*AtPgb1-*) or knocked out (*AtPgb2-*) Pgb expression were obtained in *Aarhus* University as described in Hebelstrup et al. (2006). Transgenic hybrid poplars [*Populus* x *canescens*, syn. *P. tremula* x *P. alba*, number 7171-B4, Institute de la Recherche Agronomique (INRA), Nancy, France] were generated following the protocol of Bi et al. (2015). PcPgb1+ and PcPgb2+ lines are grey poplars with overexpressing Arabidopsis class 1 PGB gene (*AtPgb1*) and Arabidopsis class 2 PGB gene (*AtPgb2*). The different tree species *Carpinus betulus* ‘Frans Fontaine’, *Fraxinus ornus ‘*Loisa Lady’, *Fraxinus pennsylvanica ‘*Summit’, *Ostrya carpinifolia*, *Celtis australis* L., *Alnus* x *spaethii* (syn. *A. japonica* x *A. subcordata*), *Alnus glutinosa* ‘Imperialis’, and *Tilia henryana* were obtained from Wilhelm Ley Baumschule (Meckenheim, Germany) and plants were 2–4 years old. These climate-resilient tree species are tested for their suitability for future urban greening in Germany (http://www.lwg.bayern.de/landespflege/urbanes_gruen/085113/index.php; Böll, 2018a; Böll, 2018b).

**Table 1:** Tree species and plants with altered PGB expression used for the different experiments in the study.

Abbreviation	Plant species	Characteristics	Number of used individual/samples
**At**	*A. thaliana* (Col-0)	Wild-type	4 (^15^NO uptake)
**AtPgb1+**	*A. thaliana* (Col-0)	Overexpressing Arabidopsis *PGB1*	4 (^15^NO uptake)
**AtPgb2+**	*A. thaliana* (Col-0)	Overexpressing Arabidopsis *PGB2*	4 (^15^NO uptake)
**AtPgb1-**	*A. thaliana* (Col-0)	Knock-down of *PGB1* (RNAi)	4 (^15^NO uptake)
**AtPgb2-**	*A. thaliana* (Col-0)	Knock-out of *PGB2*	4 (^15^NO uptake)
**Pc**	*Populus x canescens*	Wild-type	4 old, 3 young (^15^NO uptake)
**PcPgbOx1**	*Populus x canescens*	Overexpressing Arabidopsis *PGB1*	4 old, 3 young (^15^NO uptake)
**PcPgbOx2**	*Populus x canescens*	Overexpressing Arabidopsis *PGB2*	4 old, 3 young (^15^NO uptake)
**T1**	*Carpinus betulus* ´Frans Fontane´		3 (C and N content, ^15^NO uptake) 4 NO and NO_2_ deposition)
**T2**	*Fraxinus ornus* ´Loisa Lady´		3 (C and N content, ^15^NO uptake) 4 NO and NO_2_ deposition)
**T3**	*Fraxinus pennsylvanica* ´Summit´		3 (C and N content, ^15^NO uptake) 4 NO and NO_2_ deposition)
**T4**	*Ostrya carpinifolia*		3 (C and N content, ^15^NO uptake) 4 NO and NO_2_ deposition)
**T5**	*Alnus glutinosa* ´Imperialis´		4 (C and N content, ^15^NO uptake)
**T6**	*Tilia henryana*		4 (C and N content, ^15^NO uptake)
**T7**	*Alnus x spaethii*		4 (C and N content, ^15^NO uptake)
**T8**	*Celtis australis L.*		4 (C and N content, ^15^NO uptake)

### Experimental Setup and Determination of NO/NO_2_ Specific Leaf Deposition Velocities 

All experiments were performed in the phytotron chambers of Helmholtz Center in Munich, under highly controlled conditions (for a detailed description of the chambers, see Ghirardo et al., 2020). In brief, the phytotron is composed of unique climate chambers for exposure experiments of reactive gasses (Kozovits et al., 2005), and analyses of gas-exchange of CO_2_, H_2_O (Vanzo et al., 2015) under a realistic simulation of the solar radiation spectra of UV-Vis-NIR (Seckmeyer and Payer, 1993; Döhring et al., 1996; Thiel et al., 1996).

To determinate the ability of plants to emit or remove NO/NO_2_ from the atmosphere, we performed NO and NO_2_ fumigation experiments on four different tree species (*C. betulus*, *F. ornus*, *F. pennsylvanica*, and *O. carpinifolia*) under steady-state conditions by using a dynamic branch enclosure system. Experiments were repeated using different trees to obtain four replicates (n = 4). Plants were moved inside the climate chambers two days before starting the fumigation experiment, and one tree branch containing 5–8 mature leaves was enclosed the day before the NO/NO_2_ experiment. The cuvette system consisted of eight odorless polyethylene terephthalate (PET) bags (size: 60x31cm) without plasticizer (Toppits Cofresco, Minden, Germany). All the line tubes (1/4"), fittings, and T-pieces were made of the inert material polytetrafluoroethylene (PTFE). The inlet air tube was placed on the side of the stem and tightened together. Each of the eight cuvettes was continuously flushed with 1,000 ml min^-1^ of humidified (60% RH) NOx-free air (Ghirardo et al., 2020) containing ambient CO_2_ concentrations (~ 400 ppm).

The environmental conditions of the enclosed branches were: leaf temperatures of 25/12°C and relative humidity (RH) of 60/80% (light/dark); light intensities of maximum incident photosynthetically active quantum flux density (PPFD) levels of 300 μmol m-2 s-1 and a photoperiod of 14 h. Experiments started four hours after switching on the light to ensure steady-state photosynthetic conditions (Ghirardo et al., 2010; Ghirardo et al., 2014). Overall, measurements followed the experimental procedures described elsewhere (Wildt et al., 1997; Chaparro-Suarez et al., 2011), although specific leaf deposition velocities of NO/NO_2_ were determinate on tree branches. Branches of healthy trees were exposed to six different mixing ratios of NO and NO_2_ of 0, 2.5, 12.5, 25, 45, 90 ppb. Clean air enriched in NO/NO_2_ was produced by dilution steps using mass flow controllers (MKS, Andover, USA), starting from a gas cylinder containing 2% NO in N_2_ (Air Liquide, Düsseldorf, Germany), and converting 50% NO to NO_2_ using pure O_2_ and reaction chambers as previously described (Mayer et al., 2018).

NO and NO_2_ concentrations at the inlet and outlet of the cuvettes were measured online throughout all experiments by chemiluminescence technique and using an ultra-high precision and sensitive NO/NO_2_ analyzer (limit of detection <0.025 ppb; model nCLD 899Y SupremeLine, Eco Physics AG, Duernten, Switzerland). Calibration of the instrument was achieved by using N_2_ (purity 5.0) for the zero measurements and certified NO standards at 850 ppb (Air Liquide) for the span calibration.

The cuvettes were run in parallel, and NO/NO_2_ were measured sequentially by switching automatically every 9 min using an automatic multiport valve in a similar manner as described before (Ghirardo et al., 2010; Ghirardo et al., 2020). The first 8 min of measurements were used as flushing time, and the corresponding acquisition data were disregarded from the data analysis to remove any interference from the previous cuvette measurement. The last 1 min containing six measurement points (10 s integration time) were averaged and used for calculation of gas-exchange based on projected leaf area (m^2^) as previously described (Ghirardo et al., 2011). As the reference of the fumigation levels, the inlet air was measured every four cuvettes. Therefore, the entire measurement cycle through all eight cuvettes and two references took 1 h and 30 min, before switching to the next concentration and waiting another 30 min for reaching the equilibrium of NO/NO_2_ concentrations.

Fluxes (*F*) of NO (FNO) and NO_2_ (FNO_2_) (nmol m^-2^ s^-1^) were calculated following Chaparro-Suarez et al. (2011) as:

eq. (1) $$\text{F}=([{\text{C}}_{\text{out}}]-[{\text{C}}_{\text{in}}])\times \text{Q}/\text{A}$$

based on the concentration differences between the outlet ports of the branch cuvette and the inlet air reference ([*C_out_*] and [*C_in_*], respectively, in nmol mol^-1^), the enclosed project leaf area (*A*, in m^2^), and the airflow rate through the cuvettes (*Q*, in mol s^-1^). The linear relationship was calculated between FNO/FNO_2_ and the fumigated NO/NO_2_ concentration: 

eq. (2) y=kx+b

(where *x* represents the fumigated NO/NO_2_ concentration; *y*, the net exchange rates of NO (FNO) or NO_2_ (FNO_2_), *b*, the leaf emission rate of NO/NO_2_ (in nmol m^-2^ s^-1^). The deposition potential (in nmol m^-2^ s^-1^ ppb^-1^) is the slope (*k*) value of eq. 2, and the compensation point is determined as the *x* value when *y* equals zero (i.e., the NO/NO_2_ air concentrations when leaf emission equals uptake and therefore net exchange rate is zero). Deposition potentials were converted to absolute values of specific deposition velocities (s*V*_d,_ in m s^-1^) using the ideal gas equation for conditions of 1 atm and 20^o^C. Background measurements were conducted using empty cuvettes and all the data have been corrected, accordingly. All calculations were performed using data of stable leaf gas-exchange of NO/NO_2_ collected under steady-state conditions of photosynthesis. The total projected leaf area was determined from drawings of leaves on paper prior cuvette enclosure to allow an immediate harvest after the fumigation experiment.

### Determination of ^15^N Content in Leaves

30 day-old Arabidopsis, 15 day-old grey poplar (the height was around 15 cm), 40 day-old grey poplar (the height was around 50 cm), and 8 different urban tree species were used in this fumigation experiment. All plants were transferred to the climate chamber two days before fumigation. ^15^NO (99 % atom isotopic abundance) was obtained from Linde (Pullach, Germany) and diluted to 2% (v/v) with nitrogen by Westfalen AG (Münster, Germany). Fumigation with 50 ppb of ^15^NO and 50 ppb of unlabeled NO (control) was performed for 4 days. After the experiment, plant leaf material was dried at 60°C for 48 h and ground to a homogenous powder using a ball mill (Tissue Lyser II, Qiagen, Venlo, Netherlands). Aliquots of about 2 mg leaf powder was transferred into tin capsules (IVA Analysentechnik, Meerbusch, Germany). ^15^N abundance as well as N and C contents were determined with an Isotope Ratio Mass Spectrometer (IRMS, delta V Advantage, Thermo Fisher, Dreieich, Germany) coupled to an Elemental Analyzer (Euro EA, Eurovector, Milano, Italy). IRMS measurements were always be performed in comparison with one or more standards with known isotope composition in the same range of the analyzed samples. For that purpose, a laboratory standard (acetanilide), being part of every sequence in intervals, was used. A series of working standards of different weights were measured to determine the isotope linearity of the system. All lab standard measurements were also the base for the calibration of N and C content calculation. The lab standard itself was calibrated against several suitable international isotope standards (International Atomic Energy Agency, Vienna, Austria). International and suitable laboratory isotope standards were also part of every sequence to create a final correction of ^15^N covering all ^15^N results of this sequence. The accuracy of the ^15^N measurements can be described by a coefficient of variation of less than 0.5%. That of the element analyses is less than 2%.

### Modeling the NO_2_ and NO Deposition for Central Berlin

The deposition potentials measured for the four species (*C. betulus*, *F. ornus*, *F. pennsylvanica* and *O. carpinifolia*) were used to investigate the effect that planting these species may have on dry NO_x_ deposition under realistic conditions. For this purpose, we have calculated total atmospheric NO and NO_2_ deposition fluxes into the street tree foliage (F, in g m^-2^ s^-1^) within the central district of Berlin (Mitte), simplified as the product of the deposition velocity (*vd*, m s^-1^) and the NO/NO_2_ concentration (C, in g m^-3^):

eq. (3) F=vd × C

For this exercise, 78,000 trees within one district area of Berlin (Mitte, 39.47 km^2^) were considered, available from the city-tree inventory presented by Tigges et al. (2017). Species and dimension of each tree is known, indicating that the four most prominent genera in this area are *Acer* (26.2%, mostly *A. platanoides*)*, Tilia* (25.7%, mostly *T. cordata*)*, Fagus sylvatica* (17%), and *Quercus* (10.9%, mostly *Q. robur*) which together have a share of about 80%. The leaf area (LA) has been calculated using a formula based on the Beer-Lambert Law according to Nowak (1996), considering the crown dimension, which is available from the inventory.

In order to demonstrate the potential impact of the new parameters, we determined the deposition first by considering the prescribed species selection using a daytime *vd* value of 0.001 m s^-1^ for NO_2_ and 0.0001 m s^-1^ for NO for all trees (standard run). The NO_2_ value is the average of published measurements from tree species that are relevant for Central European urban areas, i.e., maple, oaks, and birches (Elkiey et al., 1982; Chaparro-Suarez et al., 2011). It is at the lower end of the range suggested by Lovett (1994) considering a wide range of plant species. For NO deposition velocity, we assumed a 10-fold smaller value as recommended by Hanson and Lindberg (1991), which is consistent with findings from Neubert et al. (1993). The simulation was carried out for the entire year 2014 using measured precipitation (data obtained from the German Weather Service) and air pollution data (from station “Mitte”, available from the BLUME network Berlin on request, https://www.berlin.de/senuvk/umwelt/umweltratgeber/de/spiu/luft.shtml). In a second simulation (scenario run), all trees of the aforementioned most abundant genera were replaced by the four species which had been investigated in the laboratory, using the experimentally measured s*V*_d_ based on projected leaf area measurements that were converted into deposition velocity (m s^-1^) to leaf canopy according to:

eq. (4) vd=sVd×LAI

Where *LAI* is the leaf area index, assumed a value of 3, commonly found for urban trees (Öztürk et al., 2015; Massetti et al., 2019). To have the largest effect, we replaced species in the order of descending *vd* for NO_2,_ which is *C. betulus* (0.933 mm s^-1^) replacing *Acer*, *O. carpinifolia* (0.666 mm s^-1^) replacing *Tilia, F. pennsylvanica* (0.600 mm s^-1^) replacing *Fagus* and *F. ornus* (0.453 mm s^-1^) replacing *Quercus* trees. All other boundary conditions were the same as in the standard run. The respective *vds* for NO are 0.568 mm s^-1^ for *C. betulus*, 0.1152 mm s^-1^ for *O. carpinifolia*, 0.184 mm s^-1^ for *F. pennsylvanica*, and 0.169 mm s^-1^ for *F. ornus*. In addition, we considered a decrease in *vd* to one-fifth of the daytime value during night as suggested by Lovett (1994), assuming the stomata to be mostly closed.

### Statistics

All experiments were performed using three or four different plants as independent replicates (n = 3-4, **Table 1**). Principal component analysis and group comparisons were done in R version 3.6.0 (R Core Team, 2019). Normality and homogeneity of variances were checked via the Shapiro-Wilk test (R Core Team, 2019) and Levene’s test with group medians (Fox and Weisberg, 2019), respectively. If both assumptions were not rejected (p > 0.05), ANOVA was applied, otherwise the non-parametric Kruskal-Wallis test. Raw p-values were Bonferroni-corrected across all variables of a data set. For posthoc analysis of significant ANOVA results, we applied Tukey’s test (Hothorn et al., 2008) to identify group differences. Letter assignment to groups was performed with multcompView (Graves et al., 2015).

## Results

### NO_x_ Deposition Velocities and ^15^NO Labeling Studies in Different Tree Species

**Figure 1:**
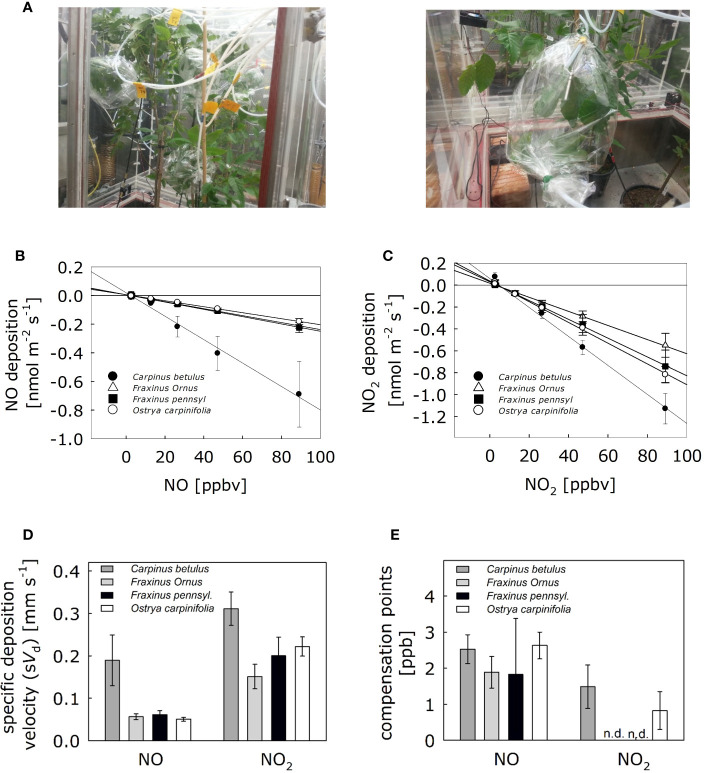
Exchange of NO and NO_2_ fluxes of four trees species resilient to heat and drought stresses suitable for urban greening. **(A)** Trees (*C. betulus*, *F. ornus*, *F. pennsylvanica* and *O. carpinifolia*) were placed into the climate chambers two days before fumigation to adapt to the environmental conditions. Fumigation experiments were performed with a dynamic branch enclosure system under steady-state conditions using NO and NO_2_. One branch was enclosed in odorless polyethylene terephthalate bags serving as cuvette system during fumigations and online measurements of NO/NO_2_. The inlet air tube was placed on the side of the stem and tightened together. Each cuvette was continuously flushed with 1,000 ml min^-1^ of humidified (60% RH) NO_x_-free air containing ambient CO_2_ concentrations (~ 400 ppm). NO_x_ was measured sequentially by switching automatically every 9 min using an automatic multiport valve. **(B)** Linear regressions of NO deposition fluxes and **(C)** NO_2_ deposition fluxes are shown. **(D)** Specific deposition velocities based on projected leaf area for each tree species was calculated from the corresponding linear regressions. **(E)** Compensation points, the NO/NO_2_ air concentrations (in ppb) when leaf emission equals uptake (i.e., net exchange rate is zero). nd, not detectable. Bars in **(C)** represents means ± SD. Experiments were replicated with four different trees. None of the NO and NO_2_ parameters in **(D)** and **(E)** showed significant differences [Kruskal-Wallis test for NO in **(D)**: p > 0.05; ANOVA for NO_2_ in **(D)** and for NO and NO_2_ in **(E)**: p>0.05; Shapiro-Wilk test: p < 0.05 for NO in **(D)**, p > 0.05 otherwise; Levene’s test: p < 0.001 for NO in **(D)**, p > 0.05 otherwise].

To determine the NO and NO_2_ specific deposition velocities to the leaf surface of *Carpinus betulus*, *Fraxinus ornus*, *Fraxinus pennsylvanica*, and *Ostrya carpinifolia*, we performed fumigation experiments and dynamic branch enclosure measurements. The experiment was performed under highly controlled environmental conditions of a phytotron. For each of the four plant species, one branch containing 5–8 mature leaves was enclosed in parallel into a respective odorless bag inside the climate chambers (**Figure 1A**). Increasing concentrations of NO and NO_2_ up to 90 ppb were applied via the inlet air, and the concentration-dependent capacity of the different tree species to remove atmospheric NO/NO_2_ was observed (**Figures 1B, C**). Then, deposition and compensation parameters were determined for NO and NO_2_. Experiments were repeated using four different trees (n = 4) per plant species. Although the different tree species did not fall into clearly distinct groups (**Supplementary Figure S1**), the foliage of *C. betulus* showed the highest NO deposition velocity, although not statistically different from that of the others (**Figure 1D**). Similary, the NO_2_ deposition velocity in *C. betulus* leaves tend to be higher compared to the other tree species (**Figure 1D**). Detected compensation points for NO were in the range of 1.8–2.6 ppb and for NO_2_ in the range of 0.8–1.5 ppb (**Figure 1E**).

**Figure 2:**
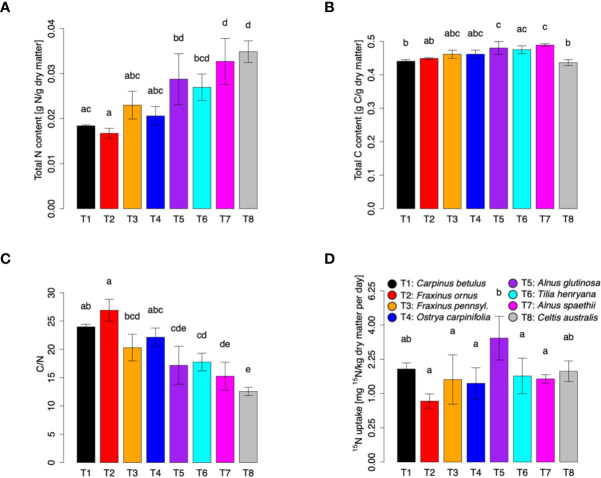
N, C, C/N ratio and ^15^N content in different tree species after fumigation with 50 ppb of ^15^NO for 5 days. Trees were exposed to 50 ppb of ^15^NO in climate chambers, and leaf samples were taken for ^15^N measurements after 4 days of treatment. Total N **(A)** and C **(B)** contents as well as ^15^N **(D)** content were determined with an Isotope Ratio Mass Spectrometer (IRMS) coupled to an Elemental Analyzer (EA). Calculated C/N ratio for each tree is shown in **(C)**. ^15^N uptake values are shown after square root transformation. Each plot represents means ± SD. Three individuals of *Carpinus betulus*, *Fraxinus ornus*, *Fraxinus pennsylvanica*, and *Ostrya carpinifolia* were measured (n = 3). Four individuals of *Celtis australis*, *Alnus spaethii*, *Alnus glutinosa*, and *Tilia henryana* were measured (n = 4). Significant species differences were observed for N, C, C/N and ^15^N (ANOVA: p < 0.01; Shapiro-Wilk test: p > 0.05; Levene’s test: p > 0.05). Different letters indicate significant differences according to Tukey’s posthoc test (p < 0.05).

To study the NO uptake capacity of tree foliage, a ^15^NO labeling experiment was performed with three trees of each species listed above. Moreover, four additional tree species (*Celtis australis*, *Alnus spaethii*, *Alnus glutinosa*, and *Tilia henryana*; four trees of each species) were included in the analysis. The trees were exposed to 50 ppb of ^15^NO for 5 days, while fumigation with 50 ppb of unlabeled NO was used as control. Then total N and C content, C/N ratio and ^15^N content were determined. Overall, the different tree species formed characteristic gradients, with *Fraxinus ornus*, *Celtis australis*, and *Alnus glutinosa* representing the extremes (**Supplementary Figure S2**). The trees differed in their total dry matter N content ranging from 0.016 g per g dry matter (*F. ornus*) to 0.036 g per g dry matter (*C. australis*) (**Figure 2A**). The total C content was in the range of 0.42 to 0.51 g per g of dry matter, with significantly larger values in both *Alnus* species than in *C. australis*, *F. ornus* and *C. betulus* (**Figure 2B**). Consequently, we observed a C/N ratio between 13 (*C. australis*) and 26 (*F. ornus*) (**Figure 2C**). The highest daily ^15^N uptake was found in *A. glutinosa* (3.6 mg per kg dry matter), followed by *C. betulus* and *C. australis*, respectively. The lowest daily uptake of ^15^N was detected in *F. ornus* (0.8 mg per kg dry matter) (**Figure 2D**).

### Modelling NO_2_ and NO Dry Deposition for Central Berlin.

**Figure 3:**
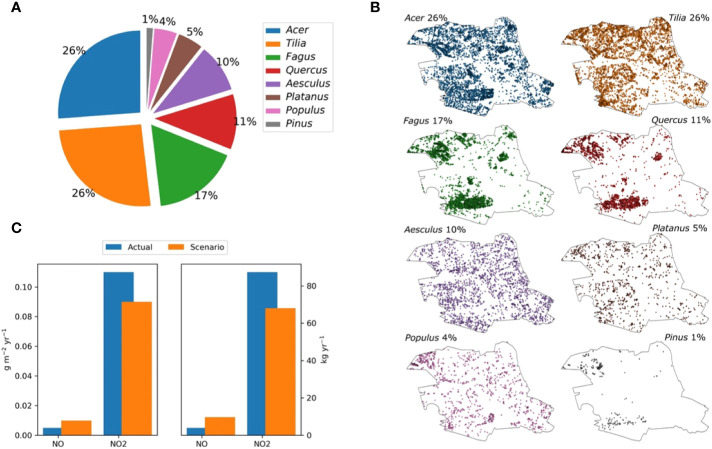
Tree population in Central Berlin and modeling of NO_x_ deposition. The calculations are done with NO_2_ and NO concentrations of the year 2014 for the central district of Berlin (Mitte). This area hosts about 78,000 trees with the species composition indicated in **(A)** and the spatial distribution depicted in **(B)**. The overall annual deposition of NO_2_ and NO per m^2^ regarding this species composition (standard) and alternative species composition (scenario) is shown in **(C)**. For the scenario, the four urban climate-resilient tree-species (*Carpinus betulus, Fraxinus ornus, Fraxinus pennsylvanica, Ostrya carpinifolia*) replaced the four actual dominant tree genera as indicated by the senate of Berlin. For parameterization, we assume that the genera can be characterized by the most abundant species in each genera group (*Acer platanoides*, *Tilia cordata*, *Fagus sylvatica*, *Quercus robur*, *Aesculus hypocastanum*, *Platanus hispanica*, *Populus nigra/alba*, and *Pinus sylvestris*).

The NO_x_ specific deposition velocities are not plant-species dependent (**Figure 1D**), concluding that all the tree species investigated are suitable for urban greening. Based on our determined NO_x_ deposition rates, we defined a tree population for the effective reduction of NO_x_ in Central Berlin. Therefore, the four dominant tree genera in this area as depicted in **Figure 3A**, which represent the standard simulation, were replaced by *C. betulus*, *F. ornus*, *F. pennsylvanica*, and *O. carpinifolia*. For discussion, we also present the distribution of each tree species within the Berlin district Mitte in **Figure 3B**. The simulations indicate that the overall pollution removal of the newly investigated species was in the same range as that of the four currently dominant genera. According to our rough estimates that assume no changes in tree dimensions or tree positions and an equal share of the abundance of the new species, the total annual NO deposition would more than double, but the NO_2_ deposition would slightly decrease by ~23% (**Figure 3C**).

### Improved NO Uptake in Phytoglobin Transgenic Arabidopsis and Poplar 

**Figure 4:**
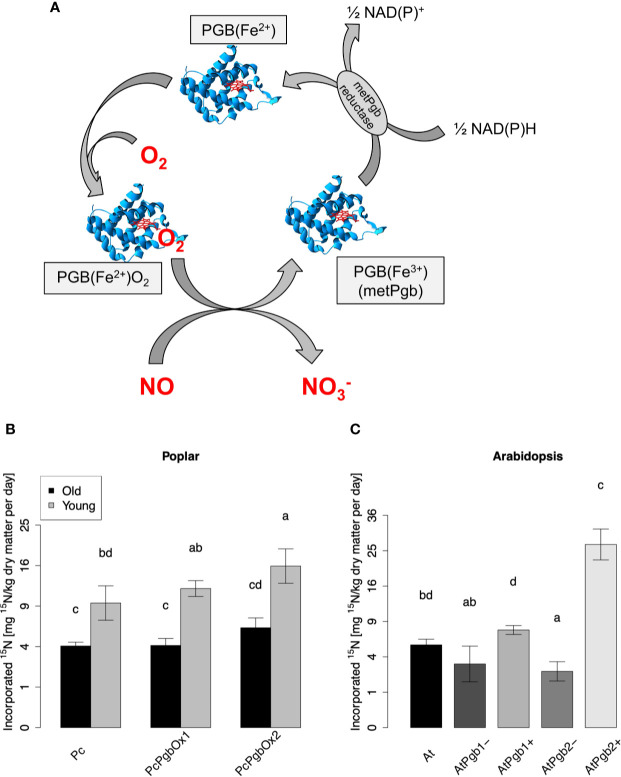
NO-fixation by plant phytoglobin. **(A)** Illustration of the biochemical NO-fixing reaction mechanism. NO is converted to nitrate (NO_3_^-^) by the oxygenated ferrous phytoglobin [PGB(Fe^2+^)], which turns to the metPgb form [PGB(Fe^3+^)]. The latter is reduced by a NAD(P)H-dependent reductase (metPGB) and then oxygenated again. **(B)** ^15^N content in transgenic poplar and **(C)** *Arabidopsis* determined 5 days after fumigation with 50 ppbv of ^15^NO. In **(B)** leaves of old (40 day-old) and young (15 day-old) poplar tree have been analyzed. Each plot depicts means ± SD. Four samples of Arabidopsis and old poplar trees were measured (n = 4) and three samples of the young poplar trees were measured (n = 3). ^15^N content values are shown after square root transformation. Different letters indicate significant differences according to Tukey’s posthoc test (p < 0.05) after significant ANOVA (p < 0.001; Shapiro-Wilk test: p > 0.05; Levene’s test: p > 0.05).

As previously reported for *Arabidopsis* and barley, PGBs are able to fix atmospheric NO into nitrogen metabolites (Kuruthukulangarakoola et al., 2017; Zhang et al., 2019). The reaction mechanism of the NO-fixation by PGB is illustrated in **Figure 4A**. To investigate if an enhanced expression of *PGBs* can enhanced NO uptake in trees, we generated transgenic grey poplar overexpressing the Arabidopsis class 1 and class 2 *PGB* gene.

The enzymatically dependent NO uptake capacity of these transgenic lines was studied in four 40-day-old trees and three 15-day-old poplars. We exposed the trees to 50 ppbv of labeled ^15^NO for 5 consecutive days under controlled environmental conditions, and we studied the ^15^N label in the harvested leaf materials. Wild type poplar of the corresponding age, as well as transgenic Arabidopsis plants with enhanced and reduced expression of *PGB* genes, were used as controls (**Figures 4B, C**). ^15^N levels in *AtPGB* overexpressing plants were higher than in the corresponding WT and *PGB* knockdown/knockout mutants (**Figure 4B**), confirming that enhanced production of the protein PGB can significantly increase the enzymatic process towards higher foliage NO uptake capacity in trees. Interestingly, we observed a higher level of ^15^N incorporation in young poplars (15 day-old poplars) in comparison to older (40 day-old poplars) plants (**Figure 4B**).

## Discussion

Our results showed that under well-watered conditions, *A. glutinosa* has the most effective NO uptake. Overall, specific deposition velocities measured in this study are in the same order of magnitude as observed in other tree species relevant for Central European urban areas such as *A. platanoides*, *A. pseudoplatanus*, *Q. robur*, *Q. petraea*, and *Betula pendula* (Elkiey et al., 1982; Chaparro-Suarez et al., 2011). However, some species seem to deviate from this average, as demonstrated in our study for *C. betulus* which at least tended to have higher s*V*_d_ for NO and NO_2_ deposition rates. Assuming a standard transformation procedure, the resulting uptake/deposition velocities were between 0.15 and 0.56 mm s^-1^ for NO and 0.45 and 0.95 mm s^-1^ for NO_2_. This strongly agrees with previous studies (Hanson and Lindberg, 1991; Hereid and Monson, 2001; Teklemariam and Sparks, 2006; Breuninger et al., 2013; Delaria et al., 2018). The reasons for the considerable difference between NO and NO_2_ are manifold: The assimilation of NO_x_ is controlled by several factors, including the resistance to the entry of NO_x_ gas molecules through the stomata and mesophyll conductance, cuticle layer, and intercellular cavity to reach the surface of mesophyll cells (Morikawa et al., 1998). Overall, NO is low soluble in water, whereas NO_2_ quickly reacts in water to form nitrate and nitrite in the apoplast (Lee and Schwartz, 1981a; Lee and Schwartz, 1981b). Because air pollutants need to go through the extracellular aqueous covering plant cell when they enter the mesophyll cells, it is logical to expect much lower deposition velocities for NO than for NO_2_. Also, the permeability of nitrate and nitrite ions through cell walls and plasma membranes (Lee and Schwartz, 1981a; Lee and Schwartz, 1981b; Ramge et al., 1993; Ammann et al., 1995), as well as the activity in the primary nitrate assimilation pathway through which NO_2_-nitrogen is reported to be metabolized do play a role in NO_x_ uptake (Rogers et al., 1979; Yoneyama and Sasakawa, 1979; Wellburn, 1990). The NO_2_ uptake by leaves of the same plant species is furthermore affected by stomatal dynamics, rate of photosynthesis, and position within the canopy (Sparks et al., 2001; Chaparro-Suarez et al., 2011). Altogether, these features can largely explain the different uptake/deposition rates for NO.

In addition to the physicochemical controls on NO/NO_2_ deposition velocities, we demonstrate that the fixation of NO is also under the control of an enzymatic process. The amount of PGB proteins and the activity of the NO-fixing machinery are important factors for an effective NO uptake and might differ between tree species. According to the biochemical activities of PGBs as NO dioxygenase [EC 1.14.12.17] (Perazzolli et al., 2004), nitrite reductase [EC 1.7.2.1] (Sturms et al., 2011a; Tiso et al., 2012; Kumar et al., 2016), and hydroxylamine reductase [EC 1.7.1.10] (Sturms et al., 2011b), they seem to be of general importance for the nitrogen metabolism. Since different PGB isoforms differ in their kinetic properties regarding oxygen and NO binding as well as NO deoxygenase activity (Smagghe et al., 2008; Calvo-Begueria et al., 2017; Eriksson et al., 2019), inducing the biosynthesis of PGBs and improving the biochemical features for NO-fixation might increase the uptake of atmospheric NO. We demonstrated that overexpression of *Arabidopsis* PGB 1 and 2 in grey poplar could indeed significantly enhance the NO uptake capacity. Therefore, producing and planting highly efficient NO_x_ removing trees, transgenic ones or after derived from “natural” selection from phenotype screening, could be a potential means to reduce the atmospheric NO_x_ level and improve air quality in urban areas.

Our modeling exercise resulted in only moderate to minor changes in overall NO_x_ removal during a full year in a Metropolitan area. Nevertheless, differences between compounds exist, indicating that the total NO_2_ deposition would only slightly decrease while NO deposition would increase by a factor of more than two, if the four dominant tree genera grown in Central Berlin would be replaced by the four tree species considered for climate change adaptation. This is partly due to the differences between the investigated species but also due to the relatively rough estimate that the standard run was based upon. Indeed, this run is parametrized with very few deposition velocity data available for the tree species populating urban areas. Besides, the scaling of specific deposition velocities based on leaf area that is also estimated with a relatively crude method includes considerable uncertainties. As a criterion for deposition removal capacity, we used stomatal conductance that is easily scalable with LAI to obtain a canopy- or regional level result (Teklemariam and Sparks, 2006; Chaparro-Suarez et al., 2011; Breuninger et al., 2013; Delaria et al., 2018). Respective functions consider crown size and competition that are estimated based on site conditions (e.g., Pace et al., 2018). Since we did this estimation based on species-specific parameterization, the differences in LAI due to the new tree species are already considered in the calculations. Nevertheless, LAI estimates may still considerably deviate from reality because the estimation method provides high uncertainty and pruning intensity as well as frequency. Thus, crown size strongly depends on the management practice of the city, which may differ with species. In addition, the stomatal dependency of gas uptake implies that growing under dry conditions substantially decrease removal capacity. Different water-use strategies thus result in differences in gaseous uptake. For example, an isohydric species such as *A. platanoides* that establishes drought resistance by closing stomata early would perform less well under medium water supply compared to an anisohydric species such as *Alnus* or *Carpinus* (Li et al., 2016). Considering this behavior, we can assume that the removal capacity of the tree species resilient to drought episodes would be higher under realistic environmental conditions and even higher under projected future climate conditions, provided the drought episodes are moderate. The picture, however, might change under more severe drought that might deplete water reservoirs completely and may induce mortality.

It is, overall, desirable to choose city tree species that have a relatively high NO and NO_2_ uptake/deposition capacity since they could provide a viable means to reduce atmospheric NO_x_ level and help meet clean air standards. The selection of appropriate tree species able to cope with increased heat and drought stress while keeping a high capacity to “clean” air may thus support urban planning strategies. Also, the NO-fixing capability of PGBs could be a valuable trait that might be increasingly applied to characterize tree species in the context of urban air quality.

## Data Availability Statement

Datasets from this study are shown as graphs in the article/**Supplementary Material**. Original data are available upon request.

## Author Contributions

JZ, AGh, AGo, RP, FB, and CL contributed conception and design of the study and performed the experiments/analysis. JZ AGh, and EG performed the statistical analysis. JZ and CL wrote the first draft of the manuscript. AGh, RG, EG, and J-PS wrote sections of the manuscript. All authors contributed to the article and approved the submitted version.

## Funding

JZ gratefully acknowledges the financial support from China Scholarship Council (CSC, File No. 201406300083).

## Conflict of Interest

The authors declare that the research was conducted in the absence of any commercial or financial relationships that could be construed as a potential conflict of interest.
