# Supplementary figures and images for: Improving Air Quality by Nitric Oxide Consumption of Climate-Resilient Trees Suitable for Urban Greening
Source: Front Plant Sci. 2020 Sep 29;11:549913. doi: 10.3389/fpls.2020.549913 (PMC7550725; doi:10.3389/fpls.2020.549913)

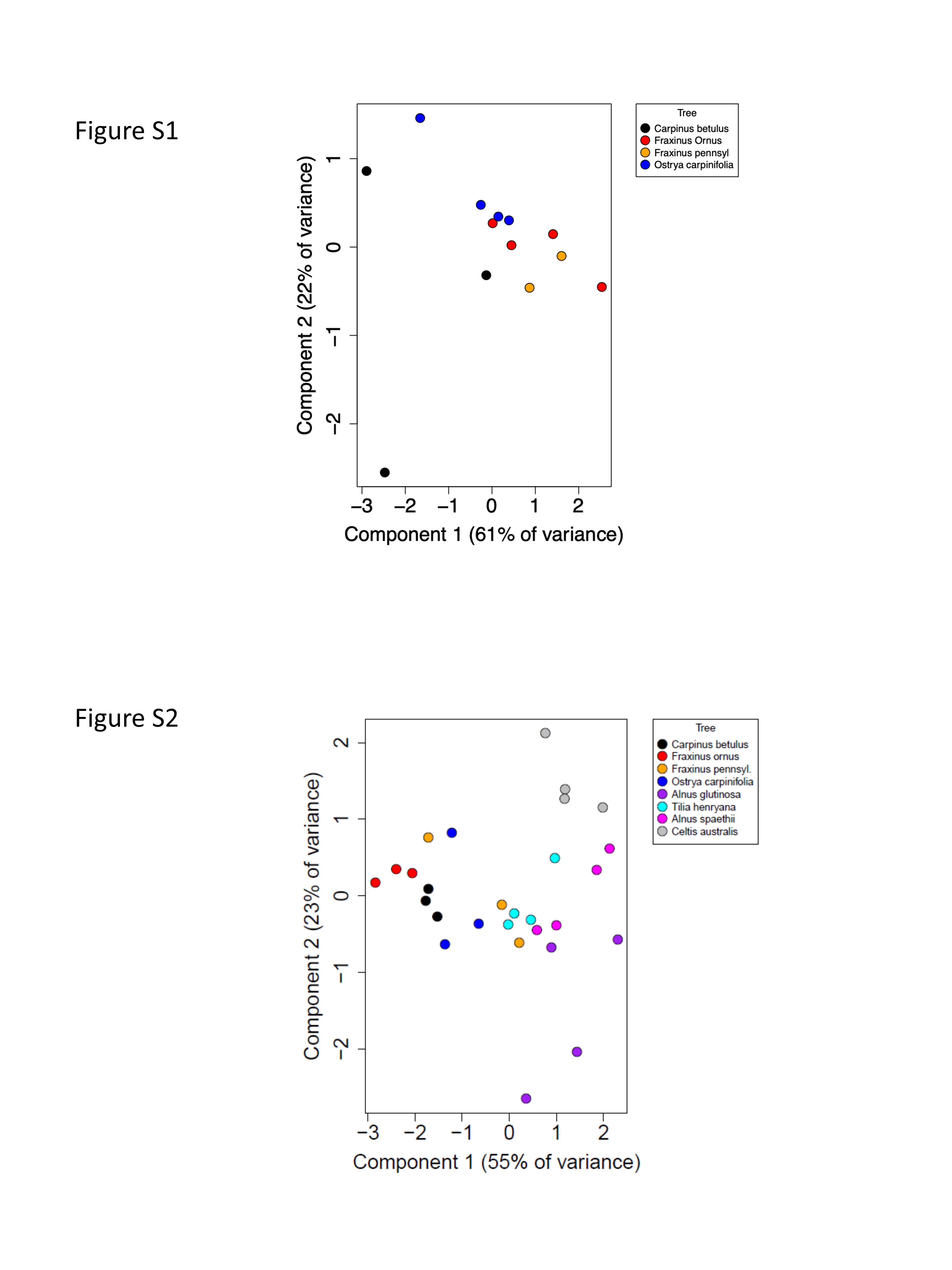

Supplement: Supplementary file 1 [file Image_1.jpeg]
